# (n, m) Distribution of Single-Walled Carbon Nanotubes Grown from a Non-Magnetic Palladium Catalyst

**DOI:** 10.3390/molecules28062453

**Published:** 2023-03-07

**Authors:** Xiaofan Qin, Dong Li, Lihu Feng, Ying Wang, Lili Zhang, Liu Qian, Wenyue Zhao, Ningning Xu, Xinyan Chi, Shiying Wang, Maoshuai He

**Affiliations:** 1 College of Chemistry and Molecular Engineering, Qingdao University of Science and Technology, Qingdao 266042, China; 2 State Key Laboratory of Rare Earth Resource Utilization, Changchun Institute of Applied Chemistry, Chinese Academy of Sciences, Changchun 130022, China; 3 Shenyang National Laboratory for Materials Science, Advanced Carbon Division, Institute of Metal Research, Chinese Academy of Sciences, Shenyang 110016, China; 4 Center for Nanochemistry, Beijing Science and Engineering Center for Nanocarbons, Beijing National Laboratory for Molecular Sciences, College of Chemistry and Molecular Engineering, Peking University, Beijing 100871, China

**Keywords:** single-walled carbon nanotube, Pd catalyst, density functional theory, interfacial formation energy, chirality distribution, bulk growth

## Abstract

Non-magnetic metal nanoparticles have been previously applied for the growth of single-walled carbon nanotubes (SWNTs). However, the activation mechanisms of non-magnetic metal catalysts and chirality distribution of synthesized SWNTs remain unclear. In this work, the activation mechanisms of non-magnetic metal palladium (Pd) particles supported by the magnesia carrier and thermodynamic stabilities of nucleated SWNTs with different (n, m) are evaluated by theoretical simulations. The electronic metal–support interaction between Pd and magnesia upshifts the *d*-band center of Pd, which promotes the chemisorption and dissociation of carbon precursor molecules on the Pd surface, making the activation of magnesia-supported non-magnetic Pd catalysts for SWNT growth possible. To verify the theoretical results, a porous magnesia supported Pd catalyst is developed for the bulk synthesis of SWNTs by chemical vapor deposition. The chirality distribution of Pd-grown SWNTs is understood by operating both Pd–SWNT interfacial formation energy and SWNT growth kinetics. This work not only helps to gain new insights into the activation of catalysts for growing SWNTs, but also extends the use of non-magnetic metal catalysts for bulk synthesis of SWNTs.

## 1. Introduction

Single-walled carbon nanotubes (SWNTs) are expected to be one of the key building blocks in the future generation of electronics and optoelectronics [1,2,3,4]. As the electrical and optical properties of SWNTs are sensitive to the SWNT chirality structure, which is denoted by chiral indices (n, m), achieving SWNTs with a narrow chirality distribution is highly desirable before incorporating SWNTs into the nanoelectronic devices [5,6,7]. In the past two decades, significant progress has been made in carbon nanotube synthesis methods including chemical vapor deposition (CVD) [8,9,10], microwave radiation [11,12], and arc discharge techniques [13], etc. Remarkably, the selective growth of SWNTs with specific structures and high purity has been realized independently by different research groups [14,15,16]. During the CVD growth process, the catalyst–SWNT interfacial interaction correlates with nanotube nucleation and carbon atom incorporation [17,18,19,20], governing the chirality distribution of the final products. To regulate the SWNT chirality distribution, a prevalent and facile method is to tune the catalyst composition. Consequently, great efforts have been made in designing different metal catalysts for selectively growing SWNTs with a narrow chirality distribution [21,22].

Besides ferromagnetic metals (including Fe, Co, and Ni) with large carbon solubility, many unconventional metal nanoparticles have also been applied for the CVD growth of SWNTs [23,24]. To remove the hurdle for investigating the intrinsic magnetic properties of SWNTs, SWNT growth from non-magnetic catalysts is indispensable. In 2006, Takagi et al. [23] demonstrated that several metal nanoparticles, including Au, Ag, Cu, Pd, and Pt, could be activated for SWNT growth after proper heat treatment in air. Later, Yuan et al. [24] reported the synthesis of horizontally aligned SWNTs on stable temperature-cut quartz substrates from various metal particles. Both works suggest that almost any metal particle with a suitable diameter could be adopted for synthesizing SWNTs. Recently, Ding et al. [25] established an SWNT formation model based on the contact angle-dependent interfacial energy of the SWNT–catalyst edge. Only when the interfacial formation energy drop counteracts the van der Waals cap–catalyst adhesion, the SWNT cap can lift off, driving the SWNT nucleation. Within the framework of heterogeneous catalysis [26], a catalytically active metal particle must fulfill the following three key parameters: decomposing the carbon precursor molecules, forming a graphitic cap on the surface, and stabilizing the SWNT end to maintain its hollow structure.

The catalytic performances of metals in growing SWNTs are predicted to be related to the *d* orbital energy [27], and a “Goldilocks zone” is established based on the energetics of a heterogeneous catalyst reaction [26]. Nørskov et al. [28] reported that adsorbed molecules preferentially interact with the *d*-states near the Fermi level of the transition metal and the carbon–metal bond strength is proportional to (1 − *f_d_*), where *f_d_* is the filling degree of the *d* band. When *f_d_* is smaller than 0.5, i.e., the *d* band is less than half-filled, carbon molecules are strongly absorbed on early transition metal surfaces and the formed strong metal–carbon bonds make the release of SWNTs extremely difficult [26]. However, the non-magnetic metal, especially the one out of the “Goldilocks zone”, is theoretically unsuitable for SWNT growth and the activation mechanisms of non-magnetic metal catalysts remain unclear. The non-magnetic metal Pd is located out of the “Goldilocks zone” [29], and the report on the growth of carbon nanotubes from the Pd catalyst is scarce [23,24], which makes the correlation establishment between Pd element and SWNT chirality distribution difficult. Recently, our group proposed to activate some noble metal catalysts by exerting metal–support interaction [30,31], which could transfer electrons from basic magnesia (MgO) support to metal clusters, shifting the originally inactive metal into the “Goldilocks zone”.

To understand the activation mechanisms of non-magnetic metals, we will choose the MgO-supported non-magnetic Pd (Pd@MgO) catalyst as the object of this work for investigating its catalytic mechanisms by using the *d*-band center model. The lower the interfacial formation energy is, the more stable the nucleated SWNT is. Experimentally, a porous MgO-supported atomically dispersed Pd catalyst is designed for bulk synthesis of SWNTs by carbon monoxide (CO) CVD. The chirality distribution of obtained SWNTs will be analyzed and compared with the theoretical results.

## 2. DFT Calculations on SWNT–Pd Interfacial Formation Energy

### 2.1. Computational Methods

Density functional theory (DFT) with the Vienna ab-initio simulation package (VASP) was applied to perform the geometric and energetic calculations [32,33,34,35]. The projector augmented wave (PAW) method was adopted to describe the interactions between ion cores and valence electrons [36,37]. The Perdew–Burke–Ernzerhof functional was used for the exchange correlation [38]. A value of 300 eV was fixed as the plane wave cutoff energy. The integration of the Brillouin zone was conducted using a 1 × 1 × 1 Monkhorst–Pack grid [39]. The convergence criteria for energy and force were set to 1.0 × 10^−5^ eV/atom and 0.05 eV/Å, respectively. Spin polarization was considered in our current study.

### 2.2. Model Construction

MgO(200) and Pd(100) surfaces were prepared by cutting MgO and Pd bulk along (200) and (100) directions. To construct a Pd@MgO catalyst, a (6 × 6) MgO(200) supercell with a three-layer slab and a (6 × 6) Pd(100) supercell with a thin slab (two layers) were selected in order to have a large enough specific surface to adsorb SWNTs. The optimized lattice of Pd@MgO was 17.19 × 17.19 Å and the mismatch was less than 5%. In this model, Pd is slightly stretched, which is beneficial to SWNT adsorption. During the optimization, the atoms in the last two layers were fixed to maintain the bulk structure, and the other atoms were allowed to fully relax. A vacuum layer of 15 Å was used along the *c* direction normal to the surface to avoid periodic interactions.

### 2.3. Interfacial Formation Energies of SWNTs on Pd@MgO Catalysts

To investigate the thermodynamic stability of the nucleated SWNTs, the interfacial formation energies of SWNTs with different (n, m) on a Pd@MgO catalyst are calculated according to the following equation [40,41]:

*E_f_* = *E*_FE_ − *E*_b_ = (0.5 × (2 × *E*_NT2_ − *E*_NT1_)) − (*E*_NT_ + *E*_Pd@MgO_ − *E*_NT@Pd@MgO_)

where *E*_FE_ and *E*_b_ are the formation energy of the free SWNT end and the SWNT–catalyst binding energy, respectively. *E*_FE_ is obtained by the equation of 0.5 × (2 × *E*_NT2_ − *E*_NT1_), in which *E*_NT1_ is the energy of a longer SWNT, and *E*_NT2_ is the energy of a shorter SWNT obtained by cutting the longer SWNT into two identical segments. Because two open ends will be formed by cutting a long SWNT, a factor of 0.5 is used in the equation. *E*_b_ can be evaluated by the equation *E*_NT_ + *E*_Pd@MgO_ − *E*_NT@Pd@MgO_, where *E*_NT_ and *E*_Pd@MgO_ are respectively the energies of separated SWNT and Pd@MgO, while *E*_NT@Pd@MgO_ is the total energy of SWNT attached to Pd@MgO. From the thermodynamic point of view, the smaller the *E_f_* value is, the more easily the SWNT is formed on the catalyst.

## 3. Results and Discussion

### 3.1. Calculation Results

**Figure 1:**
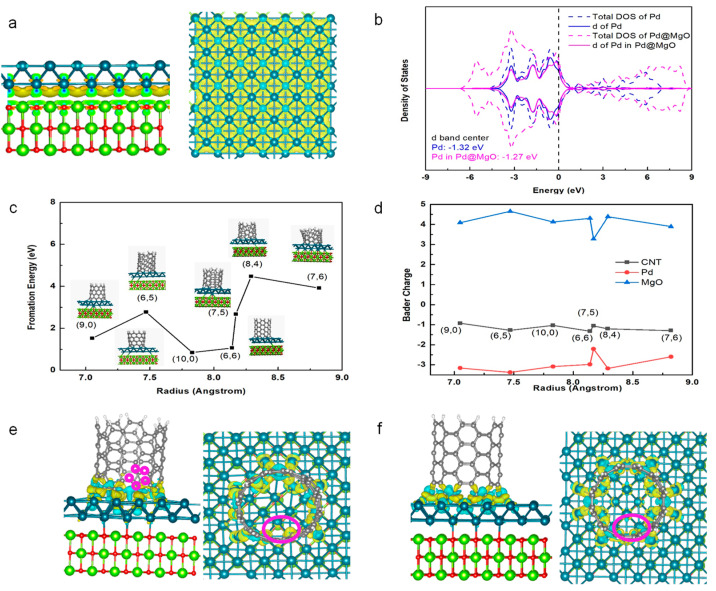
(**a**) The charge density difference for Pd@MgO. (**b**) The density of states (DOSs) of total and Pd *d* electron in Pd@MgO and Pd(100), as well as the *d*-band center. (**c**) The interfacial formation energy as the function of SWNT diameter. (**d**) The Bader charge on SWNT, MgO, and Pd. (**e**,**f**) The charge density difference between (7, 5), (6, 6) SWNT, and Pd@MgO surface, which suggests a perfect structure match and charge transfer. Isovalue = 0.005 a.u. The charge accumulation and depletion are colored respectively in yellow and cyan. Gray balls: C atoms; red balls: O atoms; green balls: Mg atoms; dark green balls: Pd atoms; white balls: H atoms. The small purple circles highlight the formed five-membered ring in (7, 5) SWNT adsorbed on Pd@MgO. The purple ellipses highlight the different charge densities induced by the five-membered ring and six-membered ring.

DFT calculations were carried out to understand the origin and underlying mechanism of SWNT growth on the late transition metal Pd regulated by the MgO substrate. In this work, we select the Pd layer model instead of the Pd nanoparticles for the following reasons. On the one hand, for the lattice well-matching systems, only a smaller supercell with fewer atoms is necessary to construct the periodic layer model. While for the nanoparticle system, a large substrate should be involved to avoid the interaction between the simulation models and images. Thus, the layer model saves lots of computational time. Furthermore, the periodic layer model is more stable and difficult to deform during the optimization process, not only maintaining the stability of the system, but also further resulting in a faster convergence than the cluster model. On the other hand, to reflect the interface effect of Pd and MgO, the double layer maybe a good choice. Since a single layer of Pd is easily deformed, while three layers of Pd will weaken the interface effect. Therefore, in order to balance the computational time and the reliability of simulation results, the double layer of metallic Pd on MgO is chosen as the model in our current study. Figure 1a,b describes the charge density difference of compound Pd@MgO and the partial density of states for Pd *d* electrons in Pd(100) and Pd@MgO. Figure 1a shows the interface of Pd accumulated the electrons from MgO and thus Pd surface is negatively charged with a value of −3.03|e|. This charge transfer process further induces the *d*-band center of Pd in Pd@MgO upshifting and closer to the Fermi level (from −1.32 to −1.27 eV, Figure 1b). The upshifting of the *d*-band center not only promotes the chemisorption and dissociation of carbon sources [42,43], but also leads to a stronger metal–carbon interaction compared to Pd(100). Therefore, metal Pd induced by MgO could be shifted into the “Goldilocks zone” [30] for SWNT nucleation and growth. Furthermore, it can be seen from Figure 1b that the total spin-up and spin-down density of state (DOS) of Pd atoms are completely symmetrical, in consistence with the fact that Pd is a non-magnetic metal catalyst. Figure 1c presents the estimated interfacial formation energies and optimized structures of various (n, m) SWNTs on Pd@MgO. All the investigated SWNTs have similar diameters (0.6–0.9 nm) but different chiral angles. Clearly, zigzag (10, 0), (9, 0) and armchair (6, 6) tubes, respectively, exhibit interfacial formation energies of 0.85, 1.53, and 1.06 eV, which are significantly lower than those of other SWNTs. The low interfacial formation energy of achiral SWNTs could be attributed to their high symmetry [19], which matches that of the underlying Pd plane. Chiral SWNTs, such as (7, 5) and (6, 5) tubes, also have low interfacial formation energies with the values of 2.68 and 2.78 eV, indicating that their nucleation on the Pd@MgO is also energetically favored. Besides, the Bader charge (Figure 1d) and charge density difference analysis (Figure 1e,f) on the configurations suggest that the charge transfers between catalyst particles and SWNTs are responsible for the strong SWNT–catalyst interactions. It is very interesting that only the (7, 5) nanotube forms a five-membered ring at the interface. Although it induces a minimum of electron transfer between Pd and MgO, the electron transfer between nanotubes and the interface is also comparable to the other tubes, accounting for the high thermodynamic stability. In addition, from a dynamic point of view, it is easy to incorporate a carbon atom into the five-membered ring and form a six-membered ring, which is conducive to continuous SWNT growth. Overall, although the strong adhesion between the SWNT and the catalyst is necessary for nucleating thermodynamically stable SWNTs [40], the kinetic factors cannot be ignored when understanding the chiral selection of SWNTs on the Pd@MgO catalyst, which will be discussed later in detail.

### 3.2. Experimental Results

**Figure 2:**
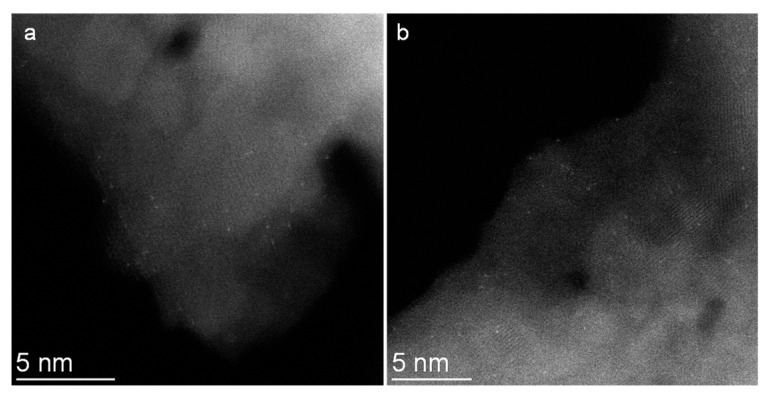
HAADF-STEM images of Pd@MgO catalyst (**a**,**b**).

In order to verify the DFT calculation results, a porous MgO-supported Pd catalyst was prepared by colloid impregnation and high-temperature annealing. Although previous reports suggested that Pd nanoparticles on flat surfaces could be applied for growing SWNTs [23,24], in our work, Pd nanoparticles directly impregnated onto porous MgO are not active for synthesizing SWNTs. As catalyst calcination has proven to be important in regulating the performances of heterogeneous catalysts [44,45], the impregnated Pd@MgO was subjected to heat treatment at 1100 °C for 4 h, which not only eliminates undesired impurities, but also helps achieve uniform distribution of metal oxides. Figure 2 shows representative aberration-corrected high-angle annular dark-field scanning transmission electron microscopy (HAADF-STEM) images of the Pd@MgO catalyst. Pd can only be observed in the form of isolated Pd atoms on the MgO surface.

**Figure 3:**
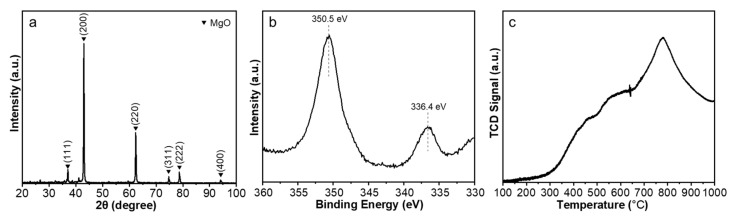
(**a**) XRD patterns, (**b**) XPS spectrum, and (**c**) H_2_-TPR profile of atomically dispersed Pd@MgO catalyst.

Figure 3a presents the X-ray diffraction (XRD) patterns of Pd@MgO catalysts over the 2θ range from 20 to 90°. Owing to the good dispersion of Pd, the diffraction peaks of PdO are not observed. Thus, the diffraction peaks at 2θ = 37.1, 43.1, 62.4, 74.8, and 78.6° can be respectively assigned to the (111), (200), (220), (311), and (222) lattice diffractions of MgO (PDF: 45-0946). The binding energies of Pd 3d in X-ray photoelectron spectroscopy (XPS) (Figure 3b) show two peaks centered at 336.4 eV and 350.5 eV, which could be correlated with the 3d_5/2_ and 3d_3/2_ of well-dispersed Pd [46]. In the catalyst, Pd atoms tend to be coordinated with oxygen atoms forming the Pd-O bond. Hydrogen temperature programmed reduction (H_2_-TPR) was adopted to evaluate the reducibility of the Pd@MgO catalyst (Figure 3c), and a full reduction of the catalyst can only be realized at a temperature higher than 800 °C, indicative of the high stability of the dispersed Pd atoms.

**Figure 4:**
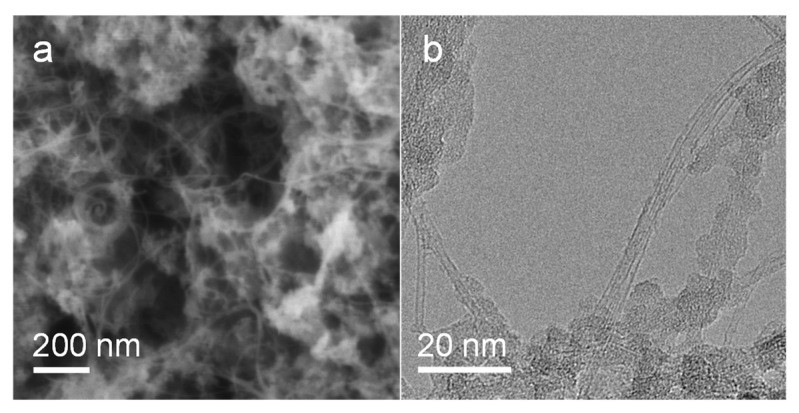
(**a**) SEM image and (**b**) TEM image of SWNTs grown from Pd@MgO catalyst at 900 °C.

Compared to supported nanoparticles, atomically dispersed metal catalysts have demonstrated superior catalytic performances in a number of heterogeneous reactions, such as selective oxidation/hydrogenation [46,47], reverse water-gas shift reaction [48], and CVD synthesis of SWNTs [30]. The atomically dispersed Pd catalyst was subjected to SWNT growth using CO as the carbon source at 900 °C, which is a bit higher than the catalyst reduction and activation temperature (Figure 3c). The morphology and structure of carbon nanotubes were analyzed by scanning electron microscopy (SEM) and transmission electron microscopy (TEM) (Figure 4). Only single-walled products were detected during the characterizations. Similar to previously reported Ru clusters [30], the reduced Pd atoms could migrate to form Pd nanoparticles during the CVD growth process (Figure 4b), which subsequently serve as the catalyst for SWNT growth. It is noted that the atomically dispersed Pd could demonstrate higher activity than Pd nanoparticles, and participate in carbon source molecule absorption and dissociation, necessary steps for SWNT nucleation. Besides, as revealed by Figure 1c, charge transfer from MgO support to Pd nanoparticles, which has previously been verified to shift inactive catalysts into the “Goldilocks zone” [30], also plays a crucial role in activating the Pd nanocatalysts.

The detailed Pd activation mechanisms could be clarified on the basis of the *d*-band center model, which is useful in understanding the catalytic activity of transition metals. As mentioned by Robertson et al. [26], a suitable catalyst for growing SWNTs should not only adsorb and dissociate the carbon precursor molecules, but also have moderate metal-carbon bonds, which allows the release and diffusion of active carbon atoms towards the open end of the nucleated SWNT. As metal Pd exhibits an electronic configuration of [Kr]d^10^, it is generally regarded as a poor catalyst for SWNT growth because of its low carbon solubility and weak interaction with CO, i.e., the carbon precursor used in the work. In the *d*-band theory, the CO chemisorption and dissociation are described by the coupling of the CO 2π* and 5σ states to the metal *d* states [42]. The strength of the bond is determined by the filling of the antibonding states, indicated by the energy of the antibonding states relative to the Fermi level. The higher in energy the *d* states are, the higher the antibonding states in energy are and the stronger the bond [43]. In short, one key parameter determining the CO–metal bond strength turns out to be the energy of the metal *d*-band center. Figure 1b clearly demonstrates that the energy of the Pd *d*-band center increases from −1.32 eV of free Pd clusters to −1.27 eV of Pd@MgO, thus promoting CO chemisorption and dissociation. As a result, the interaction with MgO support upshifts Pd *d*-band center energy, moving the Pd@MgO into the “Goldilocks zone” for SWNT synthesis.

**Figure 5:**
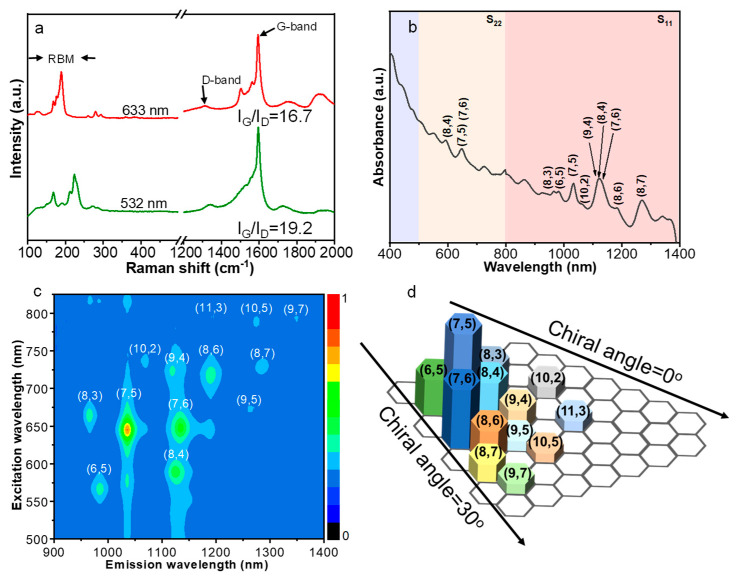
(**a**) Raman spectra of carbon nanotubes grown from Pd@MgO catalyst at 900 °C. (**b**) UV-vis-NIR absorption spectrum and (**c**) PL spectrum of SWNTs. (**d**) Chirality distribution of SWNTs deduced from PL emission intensities.

The advantages of CO over other hydrocarbon molecules in terms of growing SWNTs have been addressed previously [49,50,51]. Because of its high carburization potential, CO promotes the growth of SWNTs with a perpendicular nucleation mode. Figure 5a depicts Raman spectra acquired from as-prepared carbon nanotubes. In agreement with TEM characterization results, the relatively large intensity ratios of G/D (19.2 (532 nm), 16.7 (633 nm)) and the appearance of radial breathing modes (RBMs) suggest that the products are mainly high-quality SWNTs [52,53]. The frequencies of RBMs from two excitation laser wavelengths are mainly in the range of 160~300 cm^−1^, corresponding to SWNTs with diameters ranging from 0.7 to 1.6 nm. To evaluate the purity and content of the SWNT product, thermogravimetric analysis (TGA) was conducted on the sample synthesized at 900 °C (Supplementary Materials Figure S1). Grounded on the TGA profile, the yield of SWNTs is estimated to be lower than 4.0%. Moreover, the TGA curve shows a primary oxidation temperature of 606 °C, which is higher than the previously reported SWNTs with similar diameter distribution [54,55], confirming the superiority of the Pd@MgO catalysts. Thanks to the high quality of synthesized SWNTs on the Pd@MgO catalysts, the purified product is able to be dispersed in sodium deoxycholate solution for absorption spectroscopy characterizations (Figure 5b). Different SWNT species were clearly observed in the wavelength range of the first semiconducting exciton bands (S_11_). To overcome the overlap of absorption peaks from different (n, m) SWNTs in the absorption spectrum, photoluminescence (PL) spectroscopy mapping was applied to determine the abundance of Pd-grown SWNTs with different (n, m) (Figure 5c), based on which, the SWNT chirality map was deduced (Figure 5d) [56]. Near-armchair SWNT species, including (7, 5), (7, 6), (6, 5), and (8, 6) are the major species in the products. Besides, (2n, n) species, and those with chiral angles close to (2n, n) SWNTs, such as (8, 3), (8, 4) and (9, 4) nanotubes, also occupy a relatively large portion. In contrast, the portion of near zigzag SWNTs, such as (10, 2) and (11, 3), is relatively low.

In order to understand the SWNT chirality distribution, let us recall the DFT calculation results. From the interfacial formation energies, the SWNTs with low energy values, such as zigzag and armchair ones, are thermodynamically stable and are prone to nucleate on the Pd catalyst. However, the achiral SWNTs usually have tight contact with the underlying metal catalyst, which makes the addition of new carbon atoms to SWNT rims and SWNT growth extremely difficult [18]. Besides, the energy barriers for initiating a new carbon ring on zigzag SWNTs are usually very high [57]. Consequently, zigzag SWNTs generally suffer a very low growth rate and cannot grow long. Although the energy barrier for initiation of a new ring on armchair SWNTs is not high, incorporation of an adjacent pentagon–heptagon pair could change the chirality of an (n, n) SWNT to (n, n − 1), (n + 1, n) or (n + 1, n − 1) [58]. For instance, a (6, 6) cap could transform into (6, 5), (7, 6) or (7, 5) SWNT caps with the introduction of an adjacent pentagon–heptagon pair. Meanwhile, such near-armchair SWNTs exhibit relatively low nucleation formation energies (Figure 1c), accounting for their preferred growth and large abundance in the final product. Meanwhile, as shown in Figure 1e, there is a five-membered ring at the (7, 5) nanotube-Pd particle interface, which facilitates the easy incorporation of carbon atoms for hexagon formation and nanotube elongation.

Compared with SWNTs with large chiral angles, the (2n, n) SWNT, such as (8, 4), demonstrates a relatively high interfacial formation energy on Pd. However, the (2n, n) SWNTs have the most available kinks at the solid catalyst–SWNT interface [16,18], and thus exhibit high growth rates and possibly long lengths, responsible for their enrichment in the Pd-grown SWNTs. Similarly, the number of kinks at catalyst–SWNT interfaces for (9, 4) and (8, 3) is also large, and their fast growth rates are supposed to be correlated with their significant amount. In short, both nucleation thermodynamics and growth kinetics are responsible for the enrichment of near-armchair species and SWNTs with chiral angles close to 19.1°.

## 4. Materials and Methods

### 4.1. Preparation of Pd@MgO Catalyst

The Pd@MgO catalyst was prepared by combining the impregnation of porous MgO in Pd colloid with high-temperature calcination. The porous MgO support was obtained by annealing magnesia carbonate hydroxide at 450 °C in air. The Pd colloid was prepared by a microwave chemical reduction method. Briefly, 100 µL HCl (2 M) was added dropwise to transform 0.0018 g PdCl_2_ into H_2_PdCl_4_, which was dissolved in 9.6 mL glycol solution containing 0.0574 g poly(N-vinyl-2-pyrrolidone) (PVP, Mw = 40,000). Finally, 0.4 mL of a glycol solution of ammonia (0.2 M) was added and the solution was subjected to microwave irradiation (700 W) for 40 s.

Impregnation of porous MgO (1.0 g) in the prepared Pd colloid was carried out in 30 mL of distilled water under vigorous stirring. After drying in air at 120 °C, the catalyst was grounded into fine powders and calcined in a muffle furnace at 1100 °C for 4 h. The prepared catalyst is denoted as Pd@MgO.

### 4.2. CVD Growth of Carbon Nanotubes

An ambient pressure CVD reactor with a horizontal quartz tube (inner diameter: 40 mm) [54,59] was applied for carbon nanotube growth. After loading about 100 mg Pd@MgO catalyst into the center of the reactor, an Ar flow of 300 standard cubic centimeter per minute (sccm) was introduced to flush the reaction tube. When reaching a desired temperature of 900 °C, 300 sccm CO was switched in to replace Ar and the reaction lasted for 30 min. Finally, the furnace was cooled down to ambient temperature naturally in Ar atmosphere.

### 4.3. Characterizations of Catalyst and Carbon Nanotubes

5 mg Pd@MgO catalyst powders were firstly weighed on a glass slide with a groove of 0.5 mm. After that, the catalyst powders were flattened by a glass plate and transferred in a test chamber for XRD analysis (Bremen Germany, Bruker, D8 advance Cu Kα (λ = 0.15406 nm) radiation) with the scanning angle ranging from 20° to 90°. XPS (Waltham, MA, USA, Thermo Fisher, ESCALAB 250 Xi) was carried out to examine the Pd chemical state of the catalyst. H_2_-TPR was accomplished on a temperature-programmed chemisorption unit (Norcross, GA, USA, Micromeritic, AutoChem II 2920). The atomic structure of the Pd@MgO catalyst was characterized by HAADF-STEM (USA, Thermo Scientific, FEI-Titan Cube Themis G2 300).

Synthesized carbon nanotubes were characterized by a confocal Raman spectroscope (Wotton-under-Edge, UK, Renishaw, inVia confocal) with excitation wavelengths of 532 nm and 633 nm. SEM (Ibaraki, Japan, Hitachi, Regulus8100) was used to characterize the morphology of carbon nanotubes. Thermogravimetric analyzer (Selb, Germany, Netzsch, TG209F3) was applied to evaluate the yield and purity of carbon nanotube samples. To purify the SWNTs, the as-grown product was placed into 3 M HCl and washed thoroughly with deionized water to neutral pH. After drying, the purified nanotubes were added to a D_2_O solution containing 2 wt% sodium deoxycholate and sonicated with an 80 W output power for 2 h. The suspension was centrifuged at 100,000× *g* for 40 min to remove residual metallic particles and bundled SWNTs. The upper supernatant was characterized by UV-vis-NIR spectroscopy (Santa Clara, CA, USA, Agilent, cary5000) and PL spectroscopy (Irvine, CA, USA, HORIBA Jobin Yvon, Fluorolog-3). The purified carbon nanotubes were sonicated in acetone and dropped onto a carbon film supported by a copper grid for TEM (Tokyo, Japan, JEOL, 2100F) characterizations.

## 5. Conclusions

To conclude, DFT calculations demonstrate that the charge transfer from MgO to non-magnetic Pd clusters upshifts the Pd *d*-band center from −1.32 to −1.27 eV, intriguing the activation of the Pd@MgO catalyst for possibly growing SWNTs. Besides, the interface formation energies of different (n, m) SWNTs on Pd@MgO are investigated. The interface formation energy of (7, 5) SWNT is 2.68 eV, which is lower than that of other chiral SWNTs such as (8, 4), (7, 6), (6, 5) (4.47, 3.92, 2.78 eV, respectively), facilitating the carbon atoms to incorporate into the five-membered ring at SWNT-Pd interfaces and form (7, 5) SWNTs. Experimentally, bulk synthesis of SWNTs is realized on a well-designed Pd@MgO catalyst. Systematic characterizations reveal that near-armchair and (2n, n) SWNTs are the major species in the products, which can be explained based on the DFT calculation results and SWNT growth kinetics. This work not only sheds light on understanding the nucleation stability of different (n, m) SWNTs on the specific catalyst, but also paves an avenue for bulk synthesis of SWNTs from the non-magnetic catalyst.

## Data Availability

Not applicable.

## Supplementary Materials

The following supporting information can be downloaded at: https://www.mdpi.com/article/10.3390/molecules28062453/s1, Figure S1: TGA profile of SWNTs grown at 900 °C on Pd@MgO catalysts.
