# Supplementary figures and images for: (n, m) Distribution of Single-Walled Carbon Nanotubes Grown from a Non-Magnetic Palladium Catalyst
Source: Molecules. 2023 Mar 7;28(6):2453. doi: 10.3390/molecules28062453 (PMC10051104; doi:10.3390/molecules28062453)

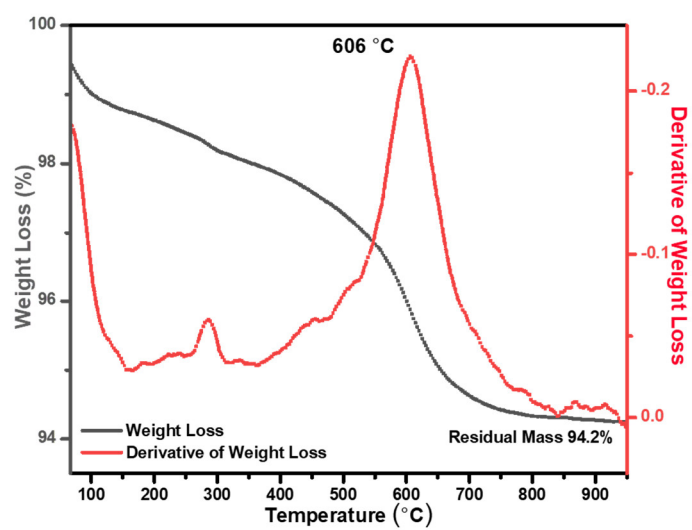

**Figure S1.** TGA profile of SWNTs grown at 900 °C on Pd@MgO catalysts.

Supplement: Supplementary file 1 [file molecules-28-02453-s001.zip › molecules-2254282-supplementary.pdf]
